# Taurine protects dopaminergic neurons in a mouse Parkinson’s disease model through inhibition of microglial M1 polarization

**DOI:** 10.1038/s41419-018-0468-2

**Published:** 2018-03-22

**Authors:** Yuning Che, Liyan Hou, Fuqiang Sun, Cong Zhang, Xiaofang Liu, Fengyuan Piao, Dan Zhang, Huihua Li, Qingshan Wang

**Affiliations:** 1 School of Public Health, Dalian Medical University, No. 9 W. Lvshun South Road, Dalian, 116044 China; 2 State Key Laboratory of Natural Products and Functions, Institute of Materia Medica, Chinese Academy of Medical Sciences & Peking Union Medical College, 100050 Beijing, China; 3 Department of Cardiology, Institute of Cardiovascular Diseases, First Affiliated Hospital of Dalian Medical University, Dalian, China

## Abstract

Microglia-mediated neuroinflammation is implicated in multiple neurodegenerative disorders, including Parkinson’s disease (PD). Hence, the modulatioein of sustained microglial activation may have therapeutic potential. This study is designed to test the neuroprotective efficacy of taurine, a major intracellular free β-amino acid in mammalian tissues, by using paraquat and maneb-induced PD model. Results showed that mice intoxicated with paraquat and maneb displayed progressive dopaminergic neurodegeneration and motor deficits, which was significantly ameliorated by taurine. Taurine also attenuated the aggregation of α-synuclein in paraquat and maneb-intoxicated mice. Mechanistically, taurine suppressed paraquat and maneb-induced microglial activation. Moreover, depletion of microglia abrogated the dopaminergic neuroprotective effects of taurine, revealing the role of microglial activation in taurine-afforded neuroprotection. Subsequently, we found that taurine suppressed paraquat and maneb-induced microglial M1 polarization and gene expression levels of proinflammatory factors. Furthermore, taurine was shown to be able to inhibit the activation of NADPH oxidase (NOX2) by interfering with membrane translocation of cytosolic subunit, p47^phox^ and nuclear factor-kappa B (NF-κB) pathway, two key factors for the initiation and maintenance of M1 microglial inflammatory response. Altogether, our results showed that taurine exerted dopaminergic neuroprotection through inactivation of microglia-mediated neuroinflammation, providing a promising avenue and candidate for the potential therapy for patients suffering from PD.

## Introduction

Parkinson’s disease (PD) is the most common neurodegenerative movement disorder characterized by progressive dopaminergic neurodegeneration in the substantia nigra pars compacta (SNpc) coupled with proteinacious inclusions composed of aggregated α-synuclein^1^. Clinical dopamine replacement therapy in patients with PD, such as levodopa, only provides symptomatic relief and has nothing to do with progressive dopaminergic neurodegeneration^2^. Although, in past decades, a numerous compounds have been found to be neuroprotective in experimental models of PD, most of them failed in translational studies^3,4^. Therefore, developing novel and effective agents aimed at arresting progressive neurodegeneration in PD is particularly urgent.

Increasing evidence has strongly suggested neuroinflammation and oxidative stress play dominant roles in driving the neurodegenerative process in neurodegenerative disorders including PD^5,6^. The activation of microglia, the innate immune cells in the brain, is the hallmark of neuroinflammation^7^. We and others previously demonstrated that SN contains high density of microglia compared with its surrounding area, whereby sustained release of high levels of cytotoxic factors contribute to the preferential damage of dopaminergic neurons^8,9^. Among the toxic factors released from activated microglia, the production of reactive oxygen species (ROS) from nicotinamide adenine dinucleotide phosphate (NADPH) oxidase (NOX2), a superoxide-producing enzyme in microglia, is an early event and plays a key role in neurotoxicity elicited by neuroinflammation^10^. Dopaminergic neurons are sensitive to inflammatory response and oxidative stress due to their low antioxidant capacity, reduced calcium buffering ability, increased accumulation of iron and high content of oxidation-prone dopamine and lipids^11–13^. Interestingly, damaged neurons can also signal microglia to trigger toxic microglial activation (defined as reactive microgliosis), resulting in additional neuron damage^5,6^. This repeating cycle of neurotoxic microglial activation is implicated in the progressive nature of PD^5,6^. Hence, the inhibition of sustained microglial activation may represent an effective therapeutic strategy to halt PD progression.

Taurine is a major intracellular free β-amino acid in mammalian tissues and mediates a myriad of physiological functions, such as neuromodulation, maintenance of calcium homeostasis, antioxidant and anti-inflammatory processes^14,15^. It was reported that the concentrations of taurine are particularly high in the SN and striatum, which plays an important role in modulating dopamine release and dopaminergic neuron activity^16^. Recent study showed that the levels of taurine in plasma of PD patients are decreased and are negatively associated with motor severity^17^, revealing a beneficial role of taurine in PD. Considering the low biosynthetic capacity of endogenous taurine, we hypothesized that supplement exogenous taurine might provide an effective approach to slow down the neurodegenerative processes of PD. Indeed, exogenous taurine has already been found to be neuroprotective against neurotoxicity induced by glutamate^18^ and hypoxic–ischemic brain damage^19^. However, the protective effects of taurine in PD remain to be investigated.

This study is therefore designed to investigate the dopaminergic neuroprotective effects and underlying mechanisms of taurine by using a mouse PD model generated by paraquat and maneb (referred to subsequently as P + M). We found that mice injected with P + M displayed progressive dopaminergic neurodegeneration, gait abnormality and α-synuclein aggregation, which was profoundly attenuated by taurine. Mechanistic study revealed that inhibition of microglial activation and M1 polarization, as well as activation of NOX2 and nuclear factor-kappa B (NF-κB) pathway contributed to taurine-afforded neuroprotection.

## Results

### P + M induce progressive dopaminergic neurodegeneration and α-synuclein aggregation in mice

**Fig. 1:**
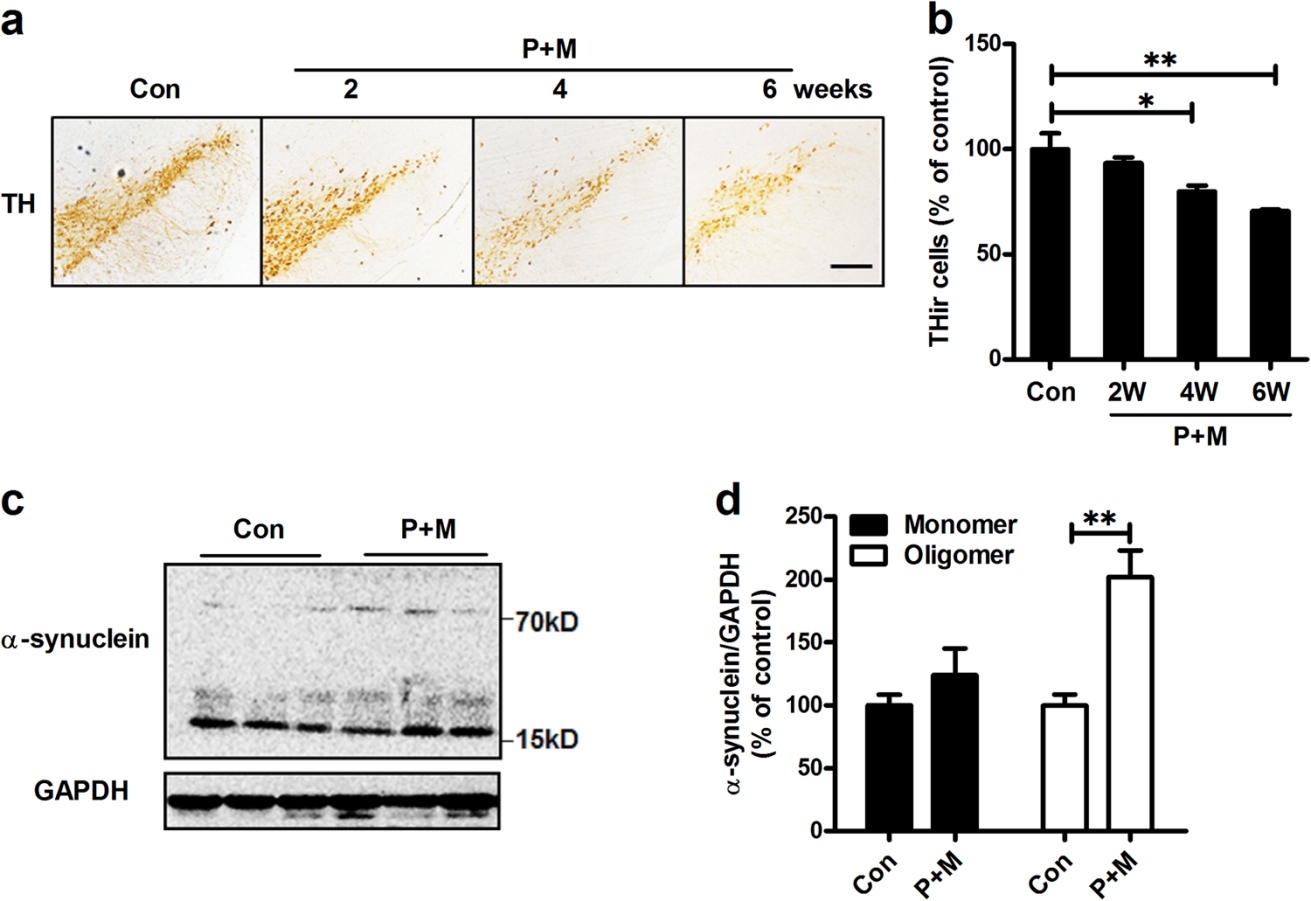
P + M induces progressive dopaminergic neurodegeneration and α-synuclein aggregation. **a** Mice were treated with P + M. After 2, 4 and 6 weeks of initial treatment, dopaminergic neurons were immunostained with anti-TH antibody and the representative images were shown. **b** The number of TH^+^ neurons in the SNpc was quantified. **c** After 6 weeks of treatment, the expression of α-synuclein in midbrain was detected by using western blot and the representative blots were shown. GAPDH was used as an internal control. **d** The band density of blots was quantified. *p< 0.05, **p < 0.01; Scale bar = 200 μm

To investigate whether P + M-intoxicated mice display progressive nature, dopaminergic neurons in the SNpc were stained by using anti-tyrosine hydroxylase (TH) antibody and TH-immunoreactive (THir) cells were counted at different time points after P + M exposure. As shown in Fig. 1a, exposed to P + M resulted in gradual loss of dopaminergic neurons (THir cells) in the SNpc of mice after 2, 4 and 6 weeks of exposure, respectively, compared with vehicle controls (Fig. 1b). To detect whether P + M-induced dopaminergic neurodegeneration is associated with α-synuclein aggregation, the expression of α-synuclein was examined in midbrains of mice after 6 weeks of treatment. The forms of α-synuclein can be induced into dimer, trimer, tetramer, fibrils and oligomers in pathological conditions, although α-synuclein is mostly monomeric under physiological condition. In order to assess the molecular forms of α-synuclein, western blot was used. Results showed that P + M mildly elevated the levels of monomeric α-synuclein, but markedly increased the expression levels of oligomeric α-synuclein (Figs. 1c, d) that had been shown to be toxic in previous studies^20,21^. Notably, although no other band was detected, we still cannot exclude the possibility that P + M could induce dimer, trimer or tetramer of α-synuclein. The reasons might be due to the low levels of these forms of α-synuclein that beyond our detect limitation in our conditions.

**Fig. 2:**
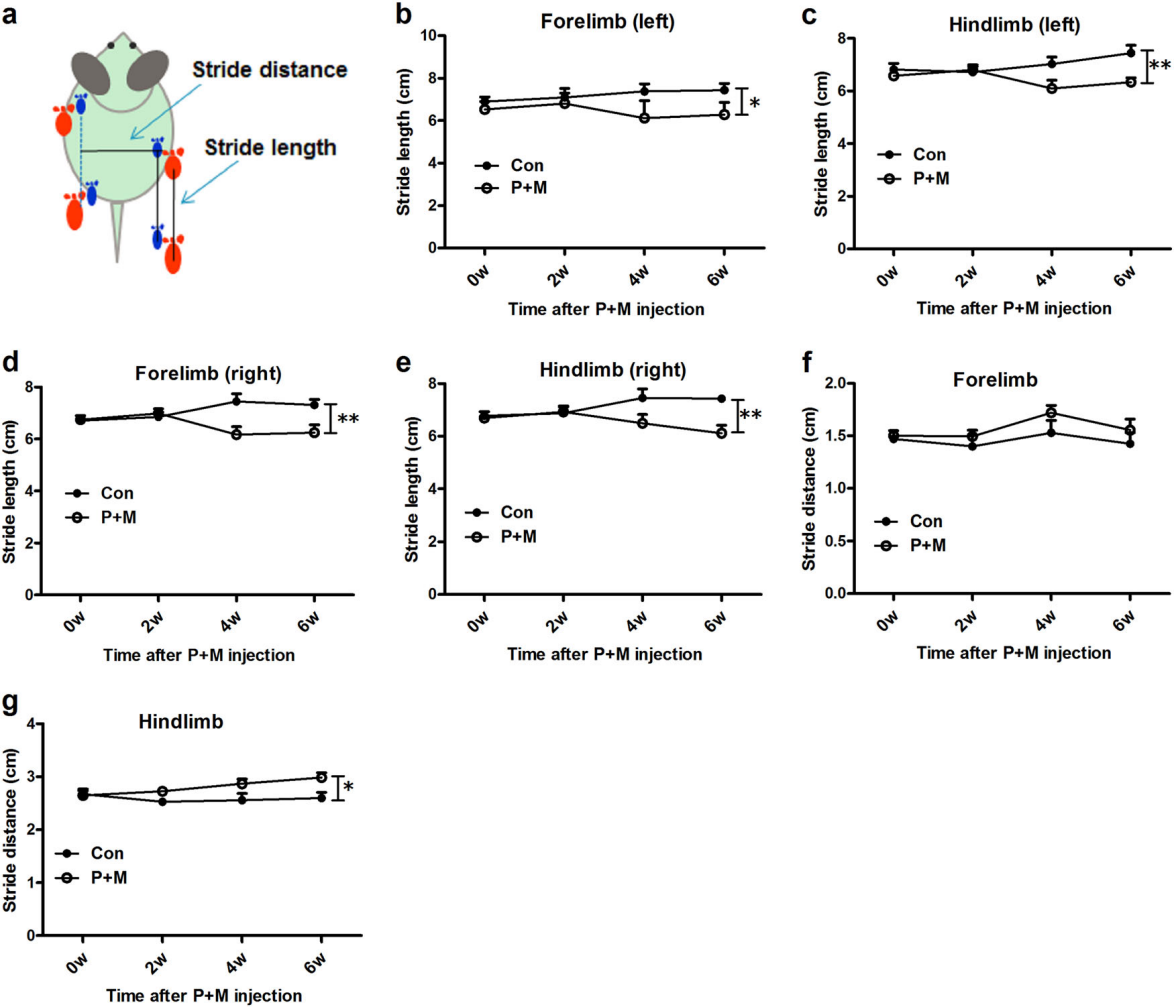
P + M induces gait abnormality in mice. **a** Schematic illustration of gait analysis measurements of stride length and stride distance was shown. **b–e** After 2, 4 and 6 weeks of initial P + M treatment, the distance between subsequent limb placements (stride length) was measured. **f**, **g** The stride distance between limb placements of the mice was detected. *p < 0.05, **p < 0.01

Gait disturbance is one of the cardinal symptoms in patients suffering PD^22^. Consistent with neuronal damage and α-synuclein aggregation, mice exposed to P + M displayed gait abnormality. Footprint analysis revealed a longer stride length of both forelimb and hindlimb in P + M-intoxicated mice after 6 weeks of treatment, compared with vehicle controls (Figs. 2a–e). In agreement, mice exposed to P + M displayed wider stride distance in hindlimb than control group, although no significant difference of stride distance in forelimb between control and P + M-treated mice was observed (Figs. 2f, g).

### Taurine ameliorates P + M-induced dopaminergic degeneration and α-synuclein aggregation in mice

**Fig. 3:**
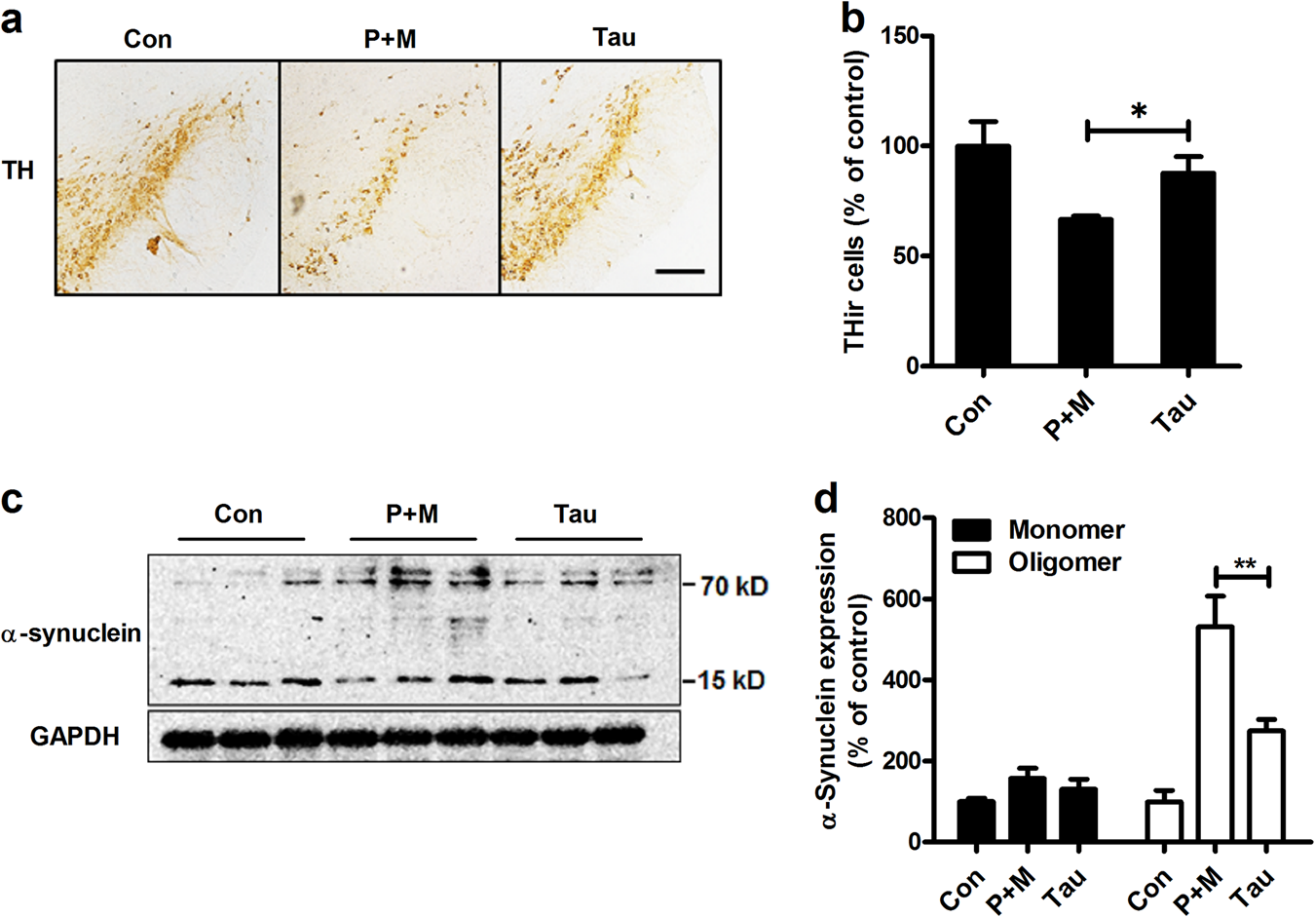
Taurine attenuates P + M-induced dopaminergic neurodegeneration and α-synuclein aggregation. **a** Taurine was administrated to mice prior to 30 min of P + M exposure. After 6 weeks of P + M treatment, dopaminergic neurons were immunostained with antibody against TH and the representative images were shown. **b** The number of TH^+^ neurons in the SNpc was quantified. **c** After 6 weeks of treatment, the expression of α-synuclein in midbrain was detected by using western blot and the representative blots were shown. GAPDH was used as an internal control. **d** The band density of blots was quantified. *p < 0.05, **p < 0.01; Scale bar = 200 μm

To determine whether taurine could protect against P + M-induced neurotoxicity, taurine (150 mg/kg) was administrated to mice for consecutive 6 weeks (twice per week). The dose of taurine was chosen based on our previous report, in which taurine alone had no significant effects on neuronal survival in mice up to 60-day treatment^23^. The number of dopaminergic neurons (THir) was compared between P + M alone and combined taurine and P + M groups. As seen in Figs. 3a, b, a higher number of THir neurons in combined taurine and P + M-treated mice than P + M alone group was observed, suggesting that taurine ameliorates dopaminergic neurodegeneration induced by P + M. No significant difference of THir neuron number was observed between vehicle and taurine alone group (Supplementary Figure S1). Consistent with dopaminergic neuroprotection, taurine also mitigated the expression of α-synuclein oligomer (Figs. 3c, d).

**Fig. 4:**
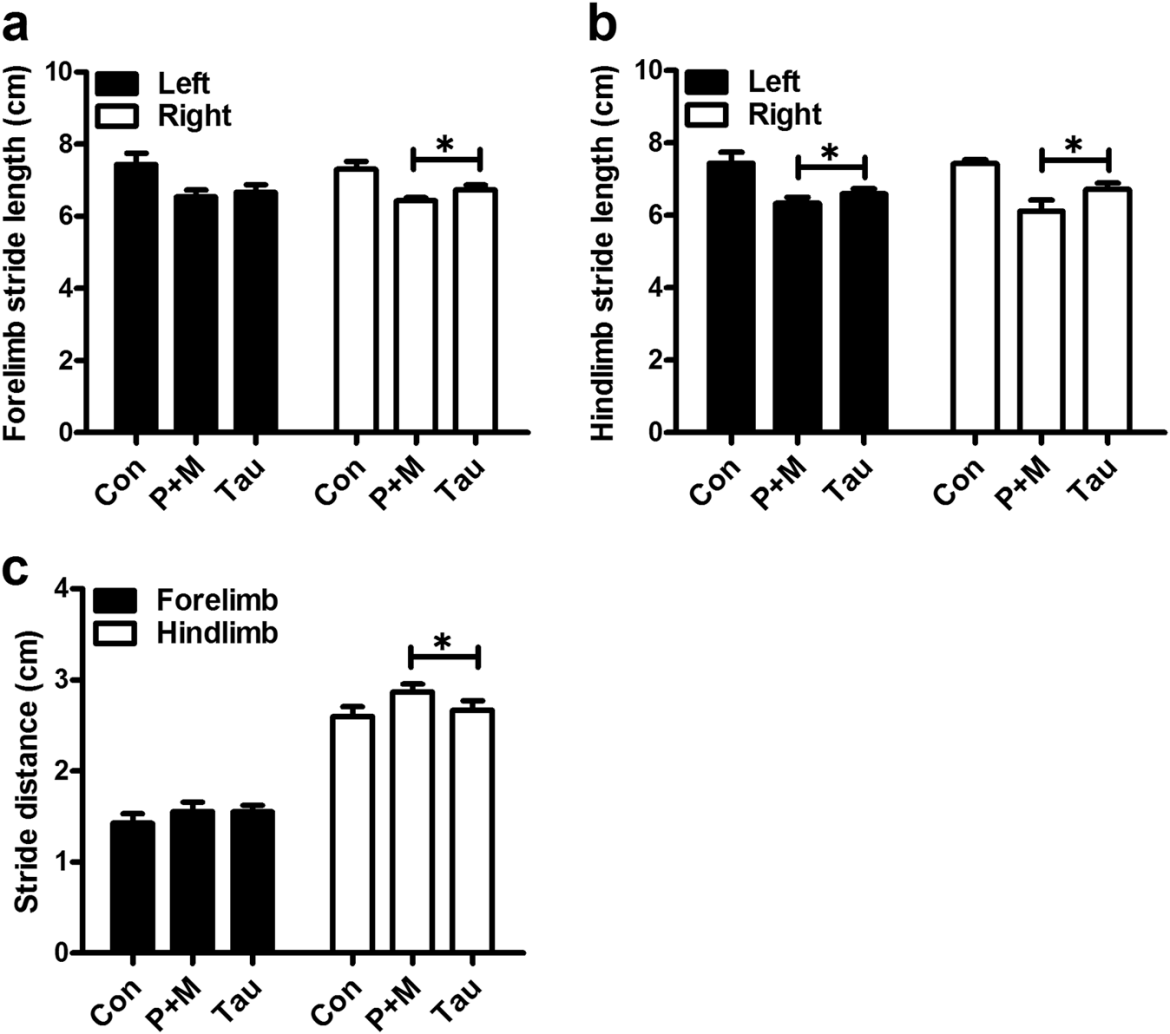
Taurine ameliorates P + M-induced motor deficits. **a**, **b** After 6 weeks of initial P + M treatment, the distance between subsequent limb placements (stride length) was measured in mice with or without taurine pre-treatment. **c** The stride distance between limb placements in P + M-intoxicated mice with or without taurine pre-treatment was detected. *p < 0.05

Taurine treatment not only showed significant neuroprotection but also displayed potent efficacy in attenuating P + M-elicited gait abnormality. Taurine treatment markedly attenuated P + M-induced reduction of stride length in both forelimb and hindlimb of mice (Figs. 4a, b). Recovered stride distance was also observed in combined taurine and P + M-treated mice compared with P + M alone group (Fig. 4c). No significant difference of stride length and stride distance was observed between taurine alone and vehicle control group (Supplementary Figure S2).

### Microglia mediate taurine-afforded dopaminergic neuroprotection

**Fig. 5:**
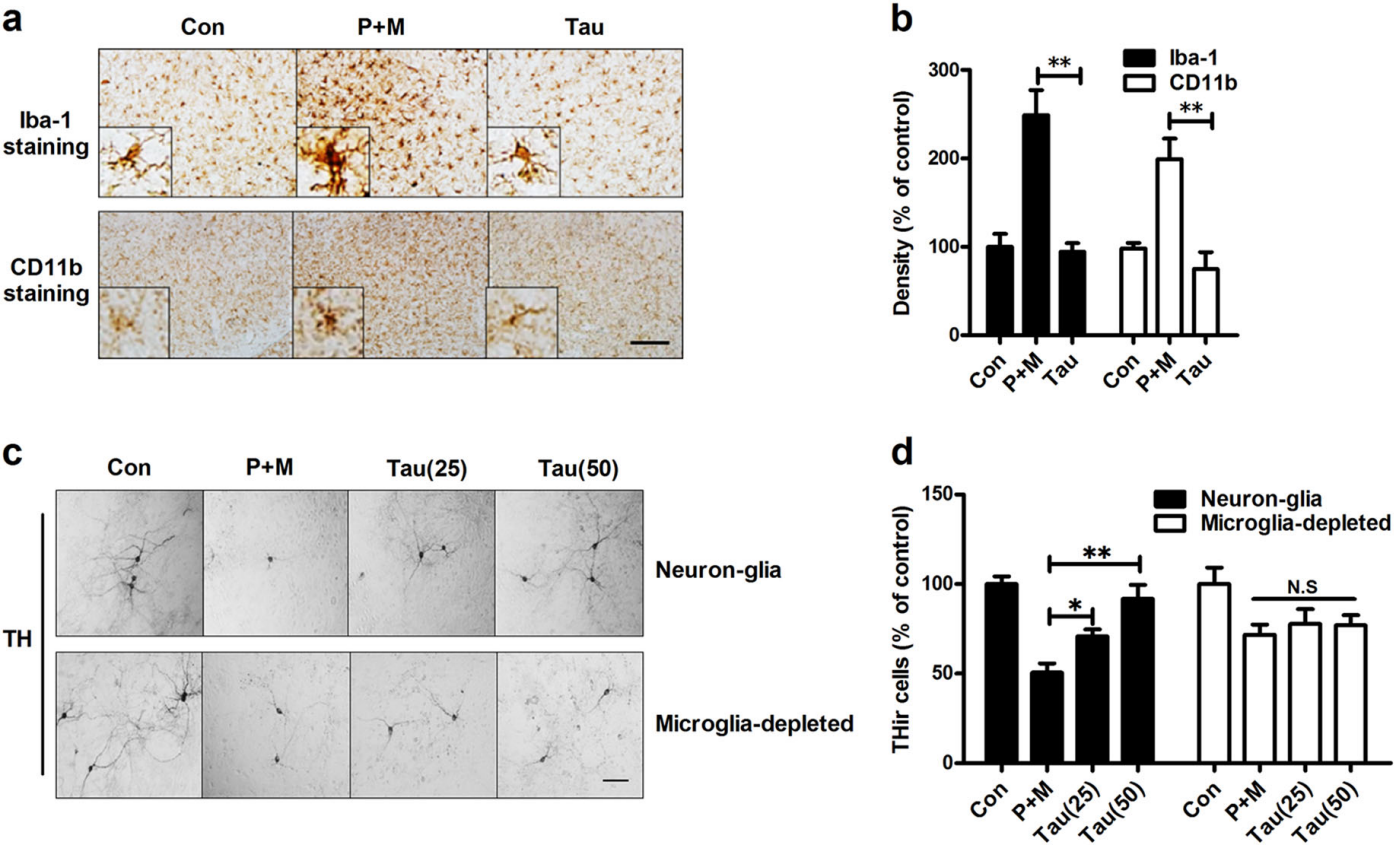
Microglia are essential for taurine-afforded dopaminergic neuroprotection. **a** After 6 weeks of initial P + M intoxication, microglial cells were immunostained with two markers, Iba-1 and CD11b, in mice with or without taurine pre-treatment and the representative images were shown. Activated microglia are characterized by enlarged cell bodies and high staining density. **b** Microglial activation was quantified by calculating the density of expression of Iba-1 and CD11b in the SN. **c** Primary midbrain neuron-glia or microglia-deleted cultures were treated with taurine (25 or 50 μM) 30 min prior to P + M lesion. After 2 days of P + M treatment, cultures were stained with antibody against TH and the representative images were shown. **d** The number of TH^+^ neurons was counted in both neuron-glia and microglia-deleted cultures. Results were expressed as a percentage of controls from three independent experiments.*p < 0.05, **p < 0.01; Scale bar = 50 μm in **a** and 100 μm in **c**

Chronic neuroinflammation mediated by microglia contributes to dopaminergic neurodegeneration in PD^5^. To determine whether the neuroprotective effects of taurine were related to inhibition of neuroinflammation, we examined the inhibitory effects of taurine on microglial activation. Microglia in the SN was stained with antibody against ionized calcium binding adaptor molecule-1 (Iba-1) and CD11b, two microglial markers. As seen in Fig. 5a, activated microglia characterized by hypertrophied morphology and intensified Iba-1 and CD11b staining were observed in the SN of mice treated with P + M. Quantitative analysis of Iba-1 and CD11b density further supported the morphological observation (Fig. 5b). Taurine treatment significantly mitigated P + M-induced microglial activation as shown by ramified morphology and reduced density of Iba-1 and CD11b staining compared with P + M alone group (Figs. 5a, b). Compared with vehicle control, taurine alone treatment had no significant effects on microglial activation (Supplementary Figure S3).

To further investigate the role of microglia in taurine-afforded neuroprotection, primary midbrain neuron-glia and microglia-depleted cultures (microglia were removed from neuron-glial cultures by L-leucine methyl ester [LME]) were prepared. We recently reported that, in our conditions, LME is capable of depleting >99.9% microglia and displays no significant effects on survival of dopaminergic neurons and functions of astrocyte^24^. The neuroprotective effects of taurine were compared between neuron-glial and microglia-depleted cultures after P + M treatment. In agreement with in vivo, P + M exposure induced a significant loss of THir neurons in neuron-glia cultures. Taurine treatment reduced P + M-induced dopaminergic neurodegeneration by showing a reduced loss of THir neurons (29.2% and 8.2% loss in 25 and 50 μM taurine + P + M-treated groups, respectively, vs. 49.4% in P + M alone group; Figs. 5c, d). By contrast, microglia depletion partially attenuated P + M-induced loss of THir neurons, which was associated with abrogation of taurine-elicited dopaminergic neuroprotection (Figs. 5c, d).

### Taurine attenuates P + M-induced microglial M1 polarization

**Fig. 6:**
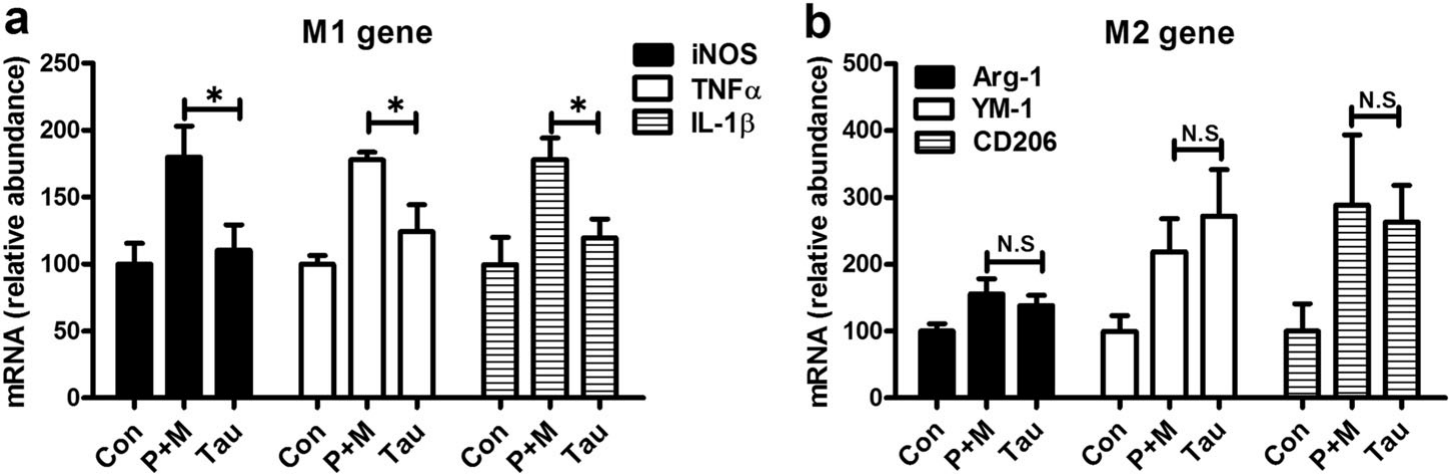
Taurine attenuates microglial M1 polarization in mice treated with P + M. **a**, **b** After 6 weeks of initial P + M intoxication, the gene expression levels of microglial M1 (iNOS, TNFα and IL-1β) and M2 (Arg-1, Ym-1 and CD206) markers were determined in midbrain of mice with or without taurine by using RT-PCR. *p < 0.05. **p < 0.01

Microglial activation can be polarized into two phenotypes, defined as “classical activation” (M1) and “alternative activation” (M2) that produce detrimental and beneficial effects, respectively^25^. To determine the mechanisms behind the inhibitory effects of taurine on microglial activation, the phenotype of microglia were initially detected in P + M-treated mice with or without taurine treatment. As seen in Figs. 6a, b, P + M injection elevated the expression levels of both M1 (inducible nitric oxide synthase [iNOS], tumor necrosis factor α [TNFα] and interleukin-1β [IL-1β]) and M2 genes (Arginase-1 [Arg-1], Ym-1 and CD206) in midbrain of mice, indicating that M1 and M2 microglia co-exist in P + M-treated mice. Interestingly, taurine treatment significantly reduced the mRNA levels of M1 genes but failed to interfere with the expression of M2 genes in P + M-treated mice. As illustrated in Figs. 6a, b, a reduced mRNA level of iNOS, TNFα and IL-1β but not Arg-1, Ym-1 and CD206 was observed in combined taurine and P + M-treated mice compared with P + M alone group.

### Taurine mitigates P + M-induced activation of NOX2

**Fig. 7:**
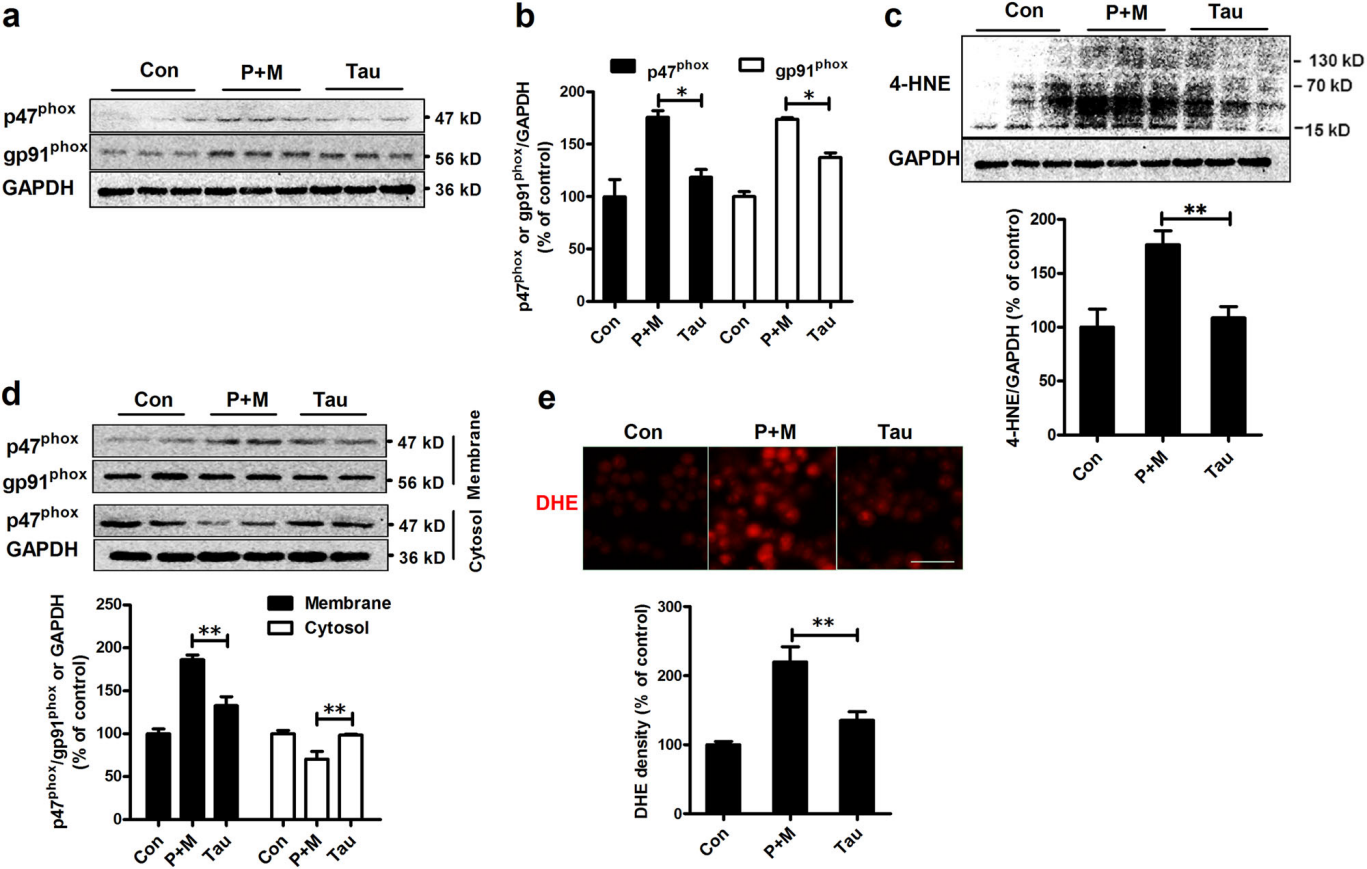
Taurine attenuates expression and activation of NOX2 induced by P + M. **a**After 6 weeks of intoxication, the levels of NOX2 subunits p47^phox^ and gp91^phox^ were determined in the midbrain of P + M-treated mice with or without taurine by western blot using specific antibodies and the representative blots were shown. **b** The band density of blots was quantified. **c** After 6 weeks of intoxication, the levels of 4-HNE were determined in the midbrain of P + M-treated mice with or without taurine by western blot and the density of blots was quantified. **d** The membrane translocation of NOX2 cytosolic subunit, p47^phox^ was detected in microglial cells using western blot and the density of blots was quantified. Gp91phox and GAPDH were used as internal membrane and cytosolic control, respectively. **e** Microglial cells were treated with P + M with or without taurine pre-treatment. The production of superoxide was assessed by DHE and the density of red fluorescence of DHE oxidation was quantified. *p<0.05, **p < 0.01

The activation of NOX2 is not only an early event during neuroinflammation but also plays a key role in modulating microglial polarization^10,26^. To investigate the effects of taurine on NOX2, the expression and activation of NOX2 were determine. As shown in Figs. 7a, b, P + M intoxication elevated the expression levels of p47^phox^ and gp91^phox^, the cytosolic and catalytic subunit of NOX2, respectively, in midbrains of mice, which was significantly reduced by taurine. Consistent with reduced NOX2 expression, taurine treatment also decreased the levels of 4-hydroxynonenal (4-HNE), a product and mediator of oxidative stress^27^, in midbrains of mice intoxicated with P + M (Fig. 7c).

To further confirm taurine was able to inhibit NOX2 activation, the membrane translocation of p47^phox^, an essential step for NOX2 activation, was investigated by using microglial cells. Western blot analysis showed that compared with vehicle controls, the levels of p47^phox^ in membrane fractions of P + M-treated microglia were significantly increased and coincidently were significantly decreased in cytosolic fractions (Fig. 7d). As seen in Fig. 7d, P + M-induced p47^phox^ membrane translocation was abrogated by taurine, suggesting that taurine inhibits NOX2 activation. The measurement of oxidation of dihydroethidium (DHE), a ROS-sensitive dye that exhibits red fluorescence through interactions with superoxide, revealed that taurine treatment also inhibited P + M-induced superoxide production by showing reduced DHE oxidation in combined taurine and P + M-treated microglia compared with P + M alone group (Fig. 7e).

### Taurine attenuates P + M-induced activation of NF-κB pathway

**Fig. 8:**
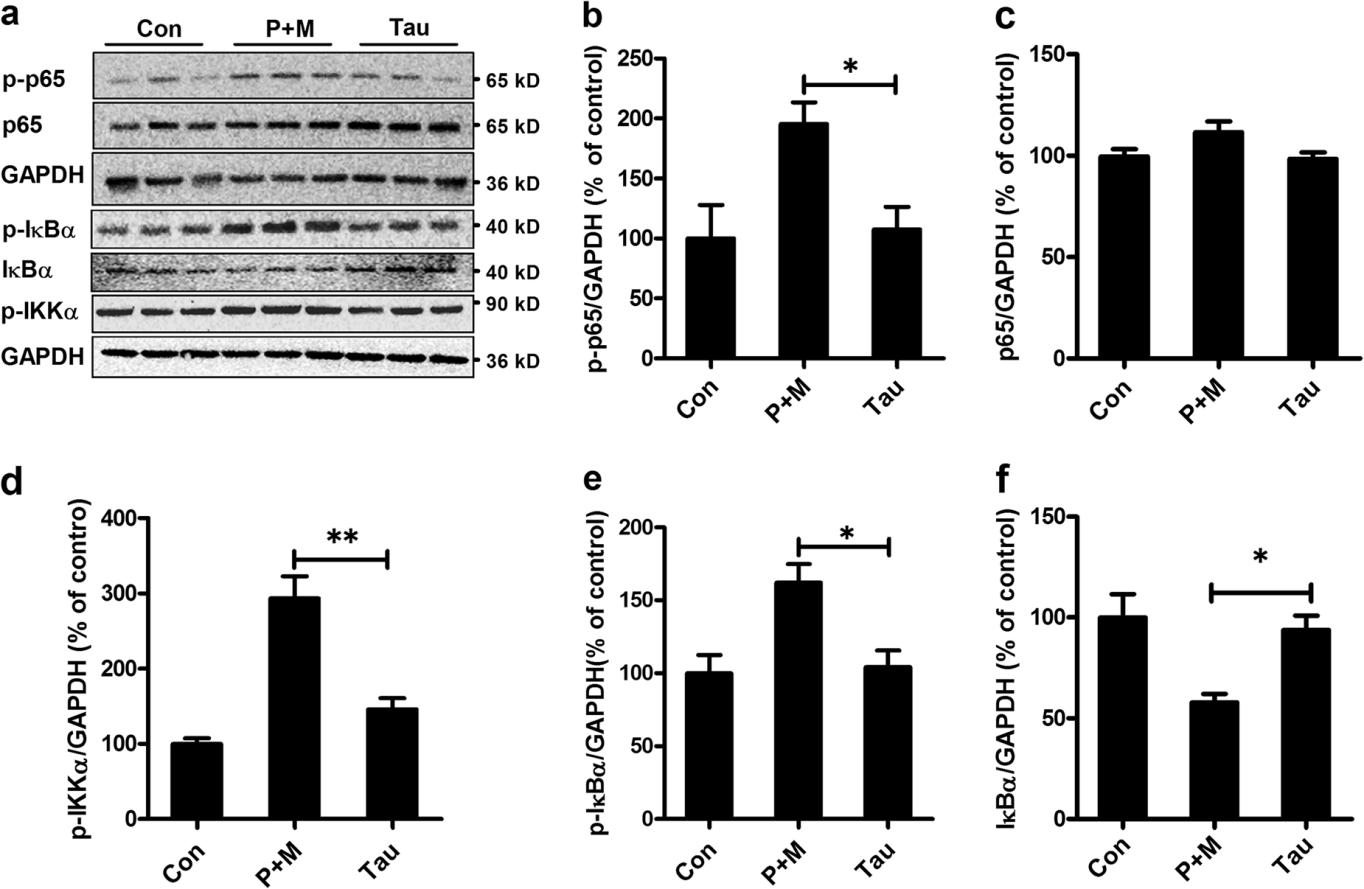
Taurine attenuates P + M-induced activation of NF-κB pathway in mice. **a** After 6 weeks of initial P + M intoxication, the levels of phosphorylated and nonphosphorylated p65, IκBα and IKKα in the midbrain of P + M-treated mice with or without taurine were determined by western blot using specific antibodies and the representative blots were shown. GAPDH was used as an internal control. **b**, **c** The density of p-p65 and total p65 blots was quantified. **d** The density of p-IKKα blots was quantified. **e**, **f** The density of p-IκBα and total IκBα blots was quantified.*p < 0.05. **p < 0.01

The NF-κB signaling pathway is reported to be essential for regulating microglial phenotypes^25^. To determine whether NF-κB signaling pathway is involved in taurine-inhibited microglial M1 polarization, the phosphorylation of p65, IκBα and IKKα, three key members of NF-κB pathway was examined. As shown in Figs. 8a–f, P + M significantly elevated the phosphorylation of p65, IκBα and IKKα, as well as degradation of total IκBα, indicating activation of NF-κB pathway. Interestingly, the activation of NF-κB signaling was not observed in P + M-treated mice when they were supplemented with taurine (Figs. 8a–f), indicating that taurine blocked P + M-induced NF-κB activation.

## Discussion

In the present study, we demonstrated that taurine provides potent beneficial effects in P + M-induced PD model. The salient features of our study are: (1) taurine ameliorated progressive dopaminergic neurodegeneration in P + M-treated mice, which was associated with improvement of motor activity of mice; (2) in addition to protecting dopaminergic neurons, taurine also attenuated P + M-induced α-synuclein oligomerization in mice; (3) taurine suppressed M1 microglial inflammatory response and deletion of microglia abolished taurine-afforded neuroprotection; (4) taurine mitigated P + M-induced activation of NOX2 and NF-κB, two key factors for microglial activation and M1 polarization.

PD is an age-related neurodegenerative disorder and affects >1.7% of population above 65. Currently, multiple therapeutic interventions have been developed to treat PD, such as dopamine replacement, deep brain stimulation and fetal brain tissue transplantation. However, all these strategies are aimed to supplement the depleted dopamine and fail to interrupt the neurodegenerative process of PD^2^. Therefore, identifying novel agents capable of arresting dopaminergic neurodegeneration gained more attention in past decades. Despite the encouraging results reported in numerous animal studies, a small percentage of these compounds have been tested in clinical trials with even fewer reaching the clinic^3,4^. The lack of suitable animal models that could recapitulate the progressive nature of PD for drug screening might be one of the reasons^28,29^. 1-Methyl-4-phenyl-1,2,3,6-tetrahydropyridine and 6-hydroxydopamine, two toxicants often used to create PD model, are known to acutely lesion dopaminergic neurons in days or even in hours^28^. In this study, we found that P + M exposure resulted in loss of dopaminergic neuron in mice in a time-dependent manner, indicating a progressive pattern of dopaminergic neurodegeneration. Additionally, mice treated with P + M also displayed elevated expression of the toxic α-synuclein oligomer. Administrating taurine attenuated dopaminergic neurodegeneration and α-synuclein oligomerization induced by P + M, which was associated with improved motor performance in mice. The protective efficacy of taurine in a progressive PD model suggests that taurine might be a promising candidate for future human study. Consistent with our findings, Alkholifi et al. reported the neuroprotective capacity of taurine in an in vitro PD model induced by rotenone^30^. The neuroprotective effects of taurine were also observed in experimental models of Alzheimer disease (AD), the most common neurodegenerative disorder. Santa-Maria et al.^31^ demonstrated that taurine could inhibit the aggregation of β-amyloid, the most component of senile plaques in AD. Taurine was also capable of inducing the synaptic potentiation and late phase long-term potentiation, which was accompanied with improved cognitive performance in experimental animals^32,33^.

Accumulating evidence suggested that chronic neuroinflammation plays a critical role in the progression of dopaminergic neurodegeneration in PD. The activation of microglia has long been observed in patients with PD, in which a vicious cycle formed between dys-regulated, over-activated microglia and damaged neurons is believed to be a driving force for the progressive nature of PD^5^. Microglial activation in response to environmental stimuli follows a polarized manner. Microglial M1 activation is characterized by the production of proinflammatory cytokines, such as TNFα, IL-1β, NO and superoxide. In contrast, the alternative activation of microglial (M2 phenotype) is hallmarked by the upregulation of anti-inflammatory and neuroprotective genes^25^. We recently reported that microglial M1 activation is associated with dopaminergic neurodegeneration in mouse PD models induced by MPTP or methamphetamine^34^. Furthermore, induction of microglial M1 polarization by lipopolysaccharide, a classic M1 microglial stimulator, damaged dopaminergic neurons in both in vitro and in vivo^35,36^. Similarly, exaggerating M1 microglial inflammatory responses by suppressing histone H3K27me3 demethylase Jumonji domain containing 3 (Jmjd3) also resulted in degeneration of dopaminergic neurons^37^. These studies suggested that M1 is the dominant phenotype for the activated microglia in the neurotoxic vicious cycle. In this study, our results showed that taurine potently inhibited P + M-induced microglial activation and gene expression of proinflammatory M1 molecules, although taurine failed to interfere with the expression of M2 genes. More importantly, depleting M1 activated microglia in P + M-treated primary cultures abolished the dopaminergic neuroprotective effects of taurine, suggesting that suppression of microglial M1 polarization might be responsible for taurine-afforded neuroprotection. Consistent with previous report^38^, no significant effects of taurine alone on microglial activation was observed. In agreement with our findings, inhibition of microglial M1 polarization by curcumin or stimulation of cannabinoid receptor-2 also improves the neurological outcome in experimental models of ischemic stroke^39^ or germinal matrix hemorrhage^40^, respectively.

The inhibitory effects of taurine on microglial M1 polarization might relate to its inactivation of NOX2 and subsequent NF-κB pathway. NOX2 is a superoxide-producing enzyme in microglia. Once activated, NOX2 not only produces extracellular superoxide but also increases intracellular ROS thought to be important secondary messengers that regulate the expression of many proinflammatory factors by activating several downstream signaling pathways including NF-κB^5,41^. ROS, especially H_2_O_2_ derived from activated NOX2 is capable of impairing NF-κB p50 function and then prolongs amplified M1 activation^42^. We recently reported that pharmacological inhibition or genetic deletion of gp91^phox^, the catalytic subunit of NOX2 abrogates NF-κB activation and gene expression levels of microglial M1 markers^29,43^, which is associated with reduction of dopaminergic neurodegeneration in experimental PD models^29,44^. In agreement with our studies, Kumar et al. also found that inactivation of NOX2 significantly reduces microglial M1 polarization in a mouse model of traumatic brain injury, leading to improvement of cognitive performance of mice^45^. These findings suggest that NOX2 could be served as a potential target to block microglial M1 polarization and subsequently disrupt the repeating vicious cycle of microglial activation, resulting in mitigation of neurodegeneration. In this study, taurine significantly reduced the expression levels of NOX2 and 4-HNE, a product and mediator of oxidative damage, in P + M-induced mouse PD model. Our in vitro finding further showed that taurine-inhibited NOX2 activation by interfering with the membrane translocation of p47^phox^. In agreement with inactivation of NOX2, reduced activation of NF-κB pathway was also observed in taurine-treated mice. Taurine alone had no significant effects on activation of NOX2 and NF-κB pathway^46,47^.

Notably, although we provided strong evidence to support a direct effect of taurine for its anti-inflammatory and neuroprotective capacity in P + M-induced PD models, taurine could also suppress inflammatory responses and neurodegeneration indirectly in vivo. Taurine is known to react with hypochlorous acid (HOCl) or hypobromous acid (HOBr), two toxic oxidants generated during inflammation, to generate the biologically active taurine chloramine (TauCl) and taurine bromamine (TauBr), respectively. Previous studies have demonstrated that taurine haloamines (TauCl, TauBr) display strong anti-inflammatory property^15,48^. On the other hand, taurine has been shown to be able to enhance the expression levels and activities of antioxidant enzymes, such as superoxide dismutase, catalase and glutathione peroxidase^15^, which might also contribute to taurine-afforded neurprotection.

### Conclusions

Altogether, this study provides convincing evidence that taurine potently reduced dopaminergic neurodegeneration and α-synuclein oligomerization through suppression of microglial M1 polarization via NOX2-NF-κB pathway in a two pesticide-induced PD model. Unlike conventional anti-inflammatory therapies that directly suppress certain proinflammatory factors, taurine not only prevented the generation of a spectrum of proinflammatory factors but also suppressed oxidative damage. Considering the safety record in human clinical trials in various pathologies including diabetes and cardiovascular disease, taurine could be a promising candidate for future clinical trials in patients of neurodegenerative diseases.

## Materials and methods

### Reagents

Paraquat, maneb and taurine were purchased from Sigma-Aldrich, Inc. (St. Louis, MO, USA). RNAiso Plus and SYBR Premix Ex Taq^TM^ II were obtained from Takara Bio Inc. (Takara, Tokyo, Japan). The membrane protein extraction kit was obtained from Beyotime (Jiangsu, China). The following primary antibodies were used: TH (EMD Millipore Corporation, Billerica, MA, USA), α-synuclein (Abcam, Cambridge, MA, USA), Iba-1 (Wako Chemicals, Richmond, VA, USA), CD11b (AbD Serotec, Raleigh, NC, USA), phosphorylated p65, p65, phosphorylated IκBα, IκBα, phosphorylated IKKα (Cell Signaling Technology, Danvers, MA, USA), 4-HNE (Abcam, Cambridge, MA, USA), p47^phox^ (EMD Millipore, Temecula, CA, USA), gp91^phox^ (BD Transduction Laboratories, San Jose, CA, USA) and GAPDH (Abcam, Cambridge, MA, USA). The BCA Protein Assay Kit was purchased from Life Technologies (Waltham, MA USA). All other chemicals were of the highest grade commercially available.

### Animal treatment

Male C57BL/6J mice (3-month old) were randomly divided into four groups, that is, control, P + M and taurine plus P + M groups (*n* = 6–9 in each group). Mice in P + M group were administrated (i.p, 5 μl/g body weight) with combined paraquat (10 mg/kg) and maneb (30 mg/kg) for consecutive 6 weeks (twice per week) according to our previous report^49^. Mice in taurine plus P + M group were received with taurine (150 mg/kg, i.p, 5 μl/g body weight) 30 min before P + M co-exposure. Mice in control group received an equivalent volume of 0.9% saline. Two days after the last taurine injection, mice were sacrificed and brains were isolated. Housing and breeding of animals were performed strictly with Dalian Medical University’s Guide for the Care and Use of Laboratory Animals. All animal procedures and their care were carried out in accordance the National Institute of Health Guide for the Care and Use of Laboratory Animals and were approved by the Institutional Animal Care and Use Committee of Dalian Medical University.

### Immunohistochemistry

For immunohistochemistry, whole brains of mice (*n* = 3–4 in each group) were removed and processed for frozen sections as described previously^29,50^ and serially sectioned at 30 μm for systematic analysis. The boundary of SN was outlined under magnification of the ×4 objective as per the atlas. The sections encompassing the entire midbrain were immunoblocked with 4–10% normal goat serum and then incubated with rabbit antibody to TH, Iba-1 or CD11b for 24 h at 4 °C. Antibody binding was visualized using a Vectastain ABC Kit and diaminobenzidine substrate. The sections were mounted permanently with Permount. Coded slides were used to ensure unbiased counting of THir neurons in every three serial section. The number of THir neurons was counted bilaterally as described previously^51^. The densities of Iba-1 and CD11b immunoreactivity were measured using ImageJ software as described previously^34^. Quantification was performed from four adjacent brain sections, spaced 120 μm apart, and was subsequently averaged for each animal. THir neuron counts and analysis of Iba-1 and CD11b staining density were performed by two individuals blind to the treatment.

### Gait analysis

Gait measurement was performed by two individuals blind to the treatment and was analyzed as previously described^52^. The forelimbs and hindlimbs of each mouse were coated with red and blue nontoxic paints, respectively. The animals were then allowed to walk along a 50-cm-long, 10-cm-wide runway into an enclosed box. All mice had three training runs and were then given one run. A fresh sheet of white paper was placed on the floor of the runway for each run. Stride length (the distance between subsequent left and right forelimb and hindlimbs) and stance width (the distance between forelimbs and hindlimbs) were measured for four to six consecutive strides. The mean value was used for subsequent analysis.

### Real-time PCR analysis

Total RNA was extracted by using RNAiso Plus and reverse transcribed with an oligodT primer according to our previous reports^49,53^. Real-time PCR amplification was performed using SYBR Premix Ex Taq^TM^ II (Takara Bio Inc., Kusatsu, Shiga, Japan) and Takara Thermal Cycler Dice^™^ Real Time System according to manufacturer’s protocols. The following primers, TNF-α (F: 5'-GACCCTCACACTCAGATCATCTTCT-3'; R: 5'-CCTCCACTTGGTGGTTTGCT-3'), iNOS (F: 5'-CTGCCCCCCTGCTCACTC-3'; R: 5'-TGGGAGGGGTCGTAATGTCC-3'), IL-1β (F: 5'-CTGGTGTGTGACGTTCCCATTA-3'; R: 5'-CCGACAGCACGAGGCTTT-3'), Arg-1 (F: 5'-GAACACGGCAGTGGCTTTAAC-3'; R: 5'-TGCTTAGCTCTGTCTGCTTTGC-3'), Ym-1 (F: 5'-AGGAAGCCCTCCTAAGGACAAACA-3'; R: 5'-ATGCCCATATGCTGGAAATCCCAC-3'), CD206 (F: 5'-AAGGAAGGTTGGCATTTGT-3'; R: 5'-CCTTTCAATCCTATGCAAGC-3') and GAPDH (F: 5'-TTCAACGGCACAGTCAAGGC-3'; R: 5'-GACTCCACGACATACTCAGCACC-3') were used. The PCR conditions were 95 °C for 10 s, 55 °C for 30 s and 72 °C for 30 s for 40 cycles. Relative mRNA gene levels were normalized to the GAPDH mRNA level and relative expression levels were determined by the comparative Ct method^49^. We set the mRNA abundance of vehicle group as 1 unit. The mRNA abundance of the other groups was expressed in reference to the vehicle controls.

### Primary cultures

Primary neuron-glia cultures were prepared as described previously^43^. In brief, cells were separated from the midbrain of embryonic day 14 ± 0.5 rats, and cultured in 5.5 × 10^5^ cells/well in poly-d-lysine-coated 24-well plates. The cultures were maintained at 37 °C in the incubator with 5 % CO_2_ and 95% air in minimum essential medium. The cultures were ready for experiments 7 days later, when the cultures became mature and stable of each cell component (~10% microglia, ~50% astrocytes, ~40% neurons and ~1% dopaminergic neurons) as described previously^52^. Microglia-depleted neuron-glia cultures were obtained by depleting microglia in neuron-glia cultures with 1.5 mM of LME 48 h after seeding (~45 % neurons and ~55 % astrocyte), as described previously^54^.

### BV2 microglial cells

The mouse microglia BV2 cell line was maintained as described previously^55^. Briefly, BV2 microglial cells were maintained at 37 °C in Dulbecco’s modified Eagle’s medium (DMEM) supplemented with 10 % fetal bovine serum, 50 U/ml penicillin, and 50 μg/ml streptomycin in a humidified incubator with 5 % CO_2_ and 95 % air. The cells were split or harvested every 3−5 days.

### Immunocytochemistry

Immunocytochemistry was performed as described previously^43,44^. Briefly, after fixation, cells were treated with 1% hydrogen peroxide. After 20 min of incubation in blocking solution (phosphate-buffered saline (PBS) containing 1% bovine serum albumin, 0.4% Triton X-100 and 4% serum), cells were incubated with anti-TH antibody for 24 h at 4 °C and followed by biotinylated secondary antibody for 2 h at room temperature. Antibody binding was visualized using a Vectastain ABC Kit and DAB substrate.

The THir cells were counted according to our previous reports^43,44^. Briefly, to quantitate cell numbers, the total number of THir neurons in a well of a 24-well plate was counted. For each experiment, two to three wells were used per treatment condition, and the results from three independent experiments were obtained.

### Membrane extraction

The membrane fractions of microglial cells were prepared using the membrane protein extraction kit as described previously^52^. Briefly, microglia were lysed in lysis buffer A provided by the kit and then subjected to Dounce homogenization (20–25 St, tight pestle A). The lysates were centrifuged at 700 × *g* for 10 min; the supernatant was collected and centrifuged at 14,000 × *g* for 30 min. The pellets were suspended using extraction buffer B and incubated for 20 min. After centrifugation at 14,000 × *g* for 5 min, the supernatant was used as membranous fraction.

### Western blot analysis

Equal amounts of protein were separated by 4–12% Bis-Tris-polyacrylamide electrophoresis gel and transferred to polyvinylidenedifluoride membranes. The membranes were incubated with primary antibody α-synuclein, 4-HNE, p47^phox^, gp91^phox^, p-p65, p65, p-IκBα, IκBα, p-IKKα and GAPDH overnight at 4 °C and followed by horseradish peroxidase-linked anti-rabbit IgG for 2 h at 25 °C. ECL reagents were used as a detection system.

### Superoxide measurement

The production of superoxide was measured by using fluorescent marker, DHE. Briefly, microglia cells were treated with P + M for 24 h with or without pre-incubation of taurine for 30 min and then were loaded with DHE (10 μM). Additional 30 min later, cells were rinsed twice with ice-cold PBS. Then, the cells were detected for superoxide generation via fluorescence microscopy (excitation 534 nm; emission 580 nm). The density of DHE oxidation was analyzed.

### Statistical analysis

All values were expressed as mean ± SEM. Differences among means were analyzed using one-way analysis of variance (ANOVA) with treatment as the independent factors. When ANOVA showed significant differences, pair-wise comparisons between means were tested by Tukey’s post hoc testing. In all analyses, the null hypothesis was rejected at the 0.05 level.

## Electronic supplementary material

Supplementary Figure Legend(DOCX 14 kb)

Supplementary Figure S1(TIF 1278 kb)

Supplementary Figure S2(TIF 1493 kb)

Supplementary Figure S3(TIF 1579 kb)
