# Supplementary material for: Taurine protects dopaminergic neurons in a mouse Parkinson’s disease model through inhibition of microglial M1 polarization
Source: Cell Death Dis. 2018 Mar 22;9(4):435. doi: 10.1038/s41419-018-0468-2 (PMC5864871; doi:10.1038/s41419-018-0468-2)
Supplement: Supplementary file 1 — Supplementary Figure Legend(DOCX 14 kb) [file 41419_2018_468_MOESM1_ESM.docx]

**Supplementary Figure Legends**

**Supplementary Figure S1. Taurine alone has no significant effects on dopaminergic neuron in the substantia nigra of mice.** (A) Taurine was administrated to mice for consective 6 weeks. The dopaminergic neurons in the substantia nigra were immunostained with antibody against TH and the representative images were shown. (B) The number of TH^+^ neurons in the subsantia nigra was quantified. Scale bar = 200 μm

**Supplementary Figure S2 Taurine alone has no significant effects on gait performance of mice.** (A-D) Taurine was administrated to mice for consective 6 weeks. The distance between subsequent limb placements (stride length) was measured in mice with or without taurine treatment. (E, F) The stride distance between limb placements in mice with or without taurine treatment was detected.

**Supplementary Figure S3.Taurine alone has no significant effects on microglia in the substantia nigra of mice.** (A) Taurine was administrated to mice for consective 6 weeks. Microglia in the subsantia nigra were immunostained with antibody against Iba-1 and the representative images were shown. (B) Microglial activation was quantified by calculating the density of Iba-1 in the subsantia nigra. Scale bar = 100 μm
