# Supplementary figures and images for: Taurine protects dopaminergic neurons in a mouse Parkinson’s disease model through inhibition of microglial M1 polarization
Source: Cell Death Dis. 2018 Mar 22;9(4):435. doi: 10.1038/s41419-018-0468-2 (PMC5864871; doi:10.1038/s41419-018-0468-2)

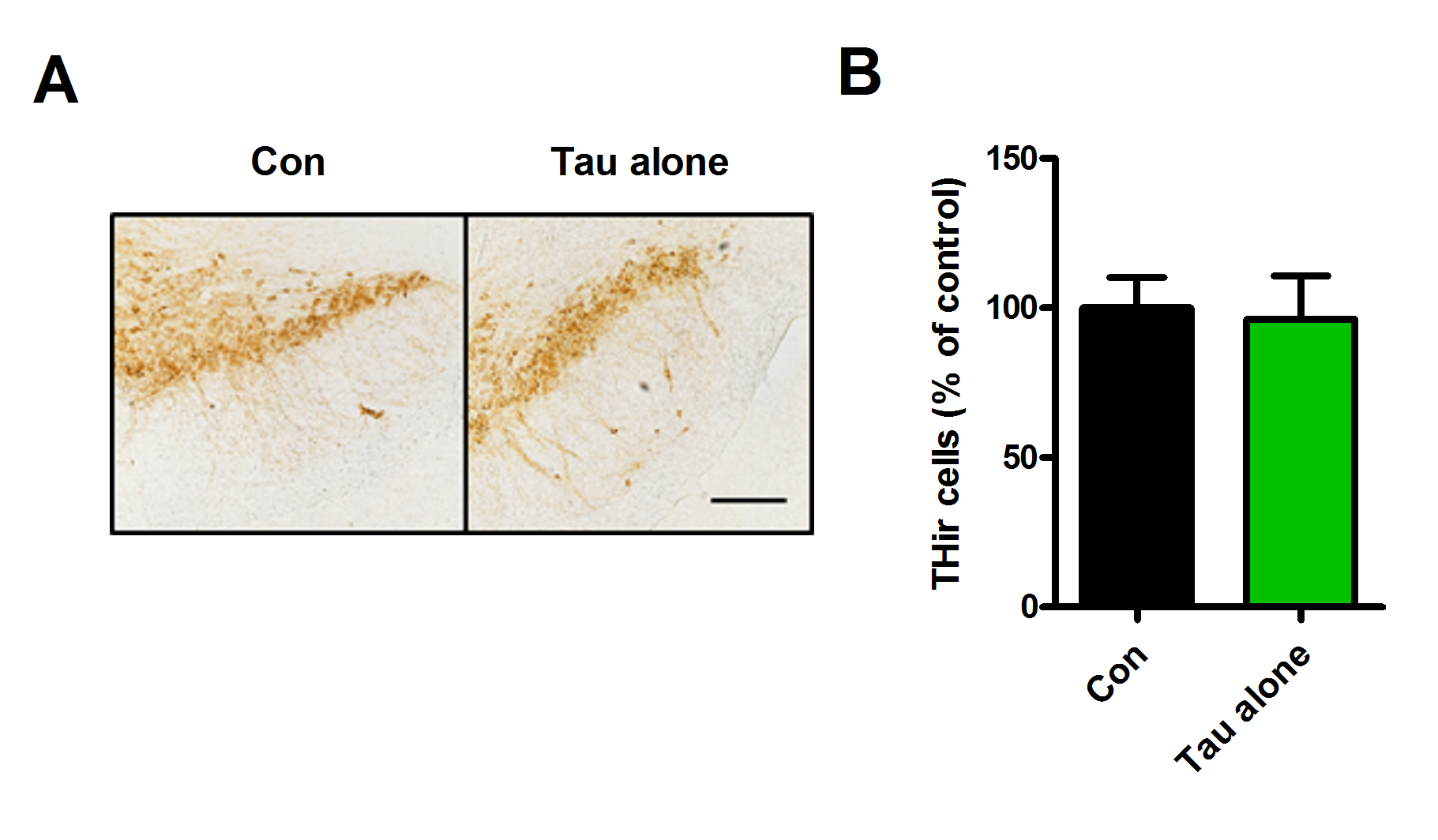

Supplement: Supplementary file 2 — Supplementary Figure S1(TIF 1278 kb) [file 41419_2018_468_MOESM2_ESM.tif]

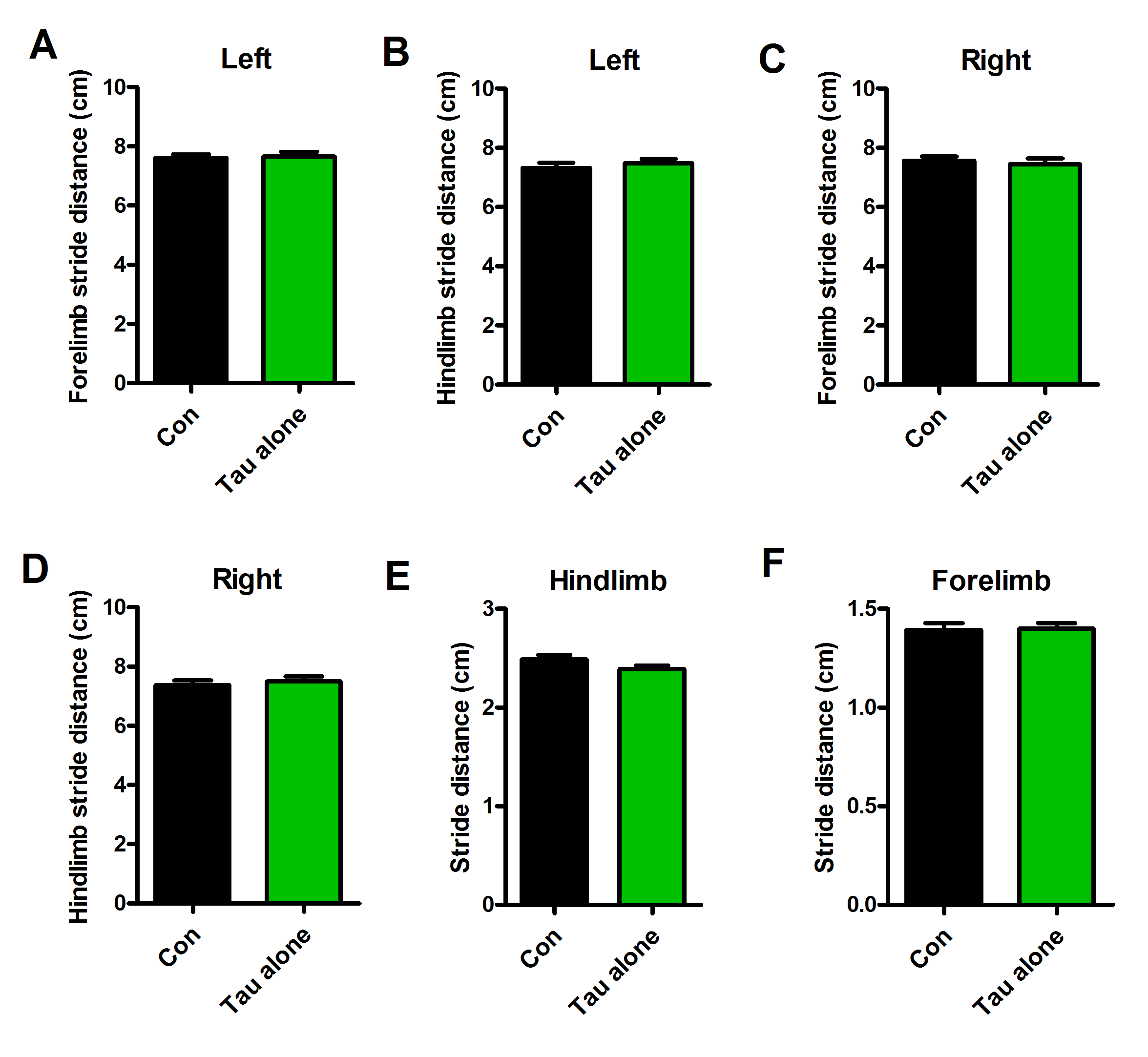

Supplement: Supplementary file 3 — Supplementary Figure S2(TIF 1493 kb) [file 41419_2018_468_MOESM3_ESM.tif]

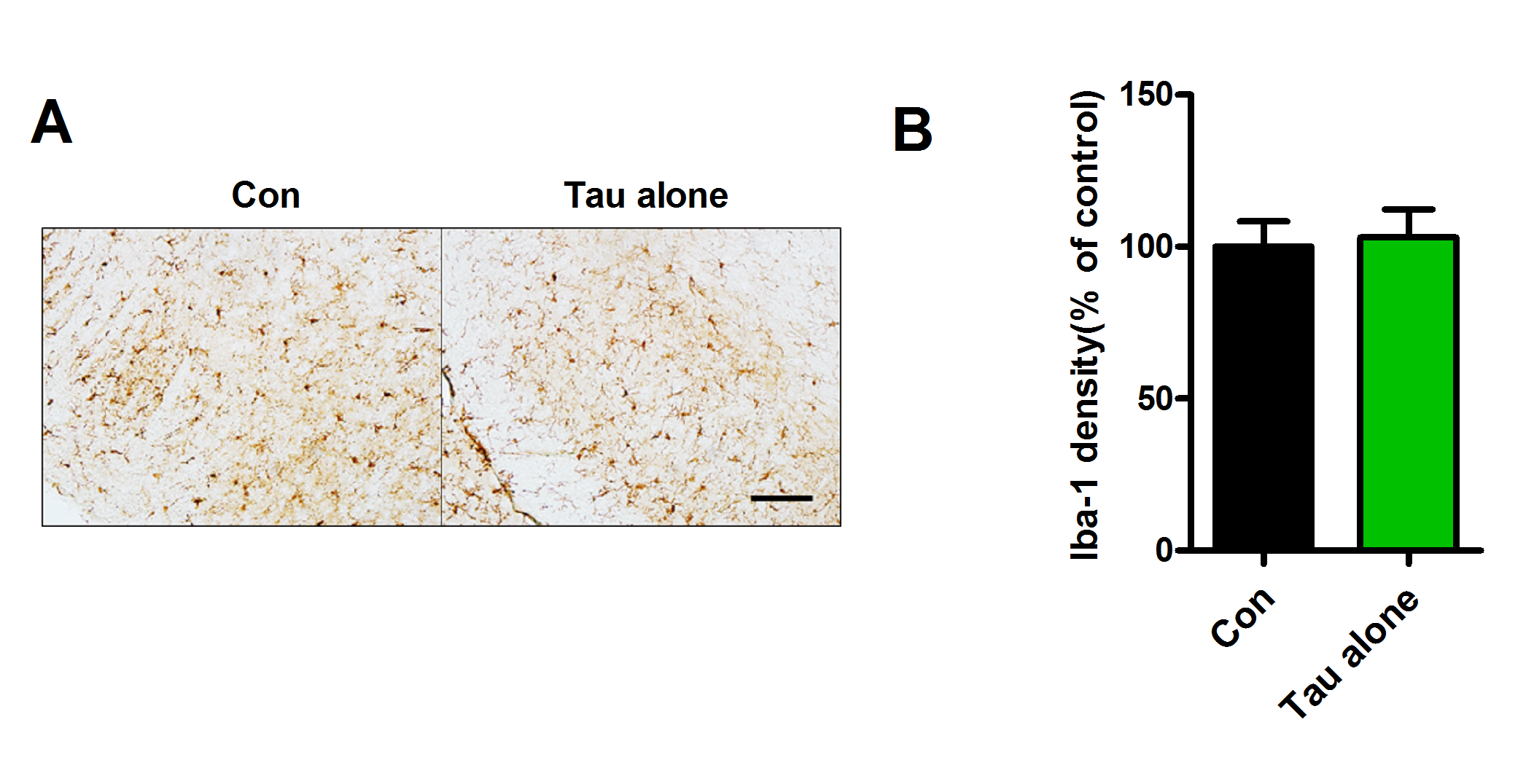

Supplement: Supplementary file 4 — Supplementary Figure S3(TIF 1579 kb) [file 41419_2018_468_MOESM4_ESM.tif]
